# A single dose of hydrocortisone does not alter interhemispheric transfer of information or transcallosal integration

**DOI:** 10.3389/fpsyt.2023.1054168

**Published:** 2023-04-18

**Authors:** Gesa Berretz, Julian Packheiser, Oliver T. Wolf, Sebastian Ocklenburg

**Affiliations:** ^1^Department of Biopsychology, Faculty of Psychology, Institute of Cognitive Neuroscience, Ruhr University Bochum, Bochum, Germany; ^2^Netherlands Institute for Neuroscience, Social Brain Lab, Amsterdam, Netherlands; ^3^Department of Cognitive Psychology, Faculty of Psychology, Institute of Cognitive Neuroscience, Ruhr University Bochum, Bochum, Germany; ^4^Department of Psychology, Medical School Hamburg, Hamburg, Germany; ^5^Institute for Cognitive and Affective Neuroscience, Medical School Hamburg, Hamburg, Germany

**Keywords:** EEG, cortisol, language, lexical decision task, asymmetry

## Abstract

Stress has been suggested as a factor that may explain the link between altered functional lateralization and psychopathology. Modulation of the function of the corpus callosum via stress hormones may be crucial in this regard. Interestingly, there is evidence that interhemispheric integration and hemispheric asymmetries are modifiable by endocrinological influences. In previous studies, our group could show an enhancing effect of acute stress on interhemispheric integration. To investigate if this effect can be attributed to an increase in the stress hormone cortisol, 50 male participants received 20 mg hydrocortisone or a placebo in a double-blind crossover design. In each test session, we collected EEG data while participants completed a lexical decision task and a Poffenberger paradigm. In the lexical decision task, we found shorter latencies of the N1 ERP component for contralateral compared to ipsilateral presentation of lexical stimuli. Similarly, we replicated the classical Poffenberger effect with shorter ERP latencies for stimuli presented in the contralateral visual field compared to the ipsilateral visual field. However, no effect of cortisol on latency differences between hemispheres could be detected. These results suggest that a temporary increase in cortisol alone might not be enough to affect the interhemispheric transfer of information via the corpus callosum. Together with previous results from our group, this suggests that chronically elevated stress hormone levels play a more central role in the relationship between altered hemispheric asymmetries and a variety of mental disorders.

## 1. Introduction

Stress hormones have been proposed to play an integral part in the development and maintenance of several mental disorders (1–3). Many patients with neurodevelopmental and mental disorders display alterations in typical asymmetry patterns as well as the regulation of stress hormones (4, 5). This suggests that there is a connection between reduced functional hemispheric asymmetries (FHAs) and chronically increased circulating stress hormones. However, it is not clear if a similar association between reduced FHAs and a short-term increase in cortisol due to acute stress exists. While changes in FHAs and interhemispheric communication due to fluctuations of sex hormone levels have been studied for several decades (6, 7), the influence of other hormones on the interaction of the hemispheres has only recently come into focus (8). Specifically, whether this connection is causal or purely correlational in nature remains to be investigated.

The corpus callosum as the main commissure in the human brain is integral to the exchange and integration of information between both hemispheres and for the emergence of hemispheric asymmetries (9). While its fibers are glutamatergic and thus excitatory in nature, callosal fibers mainly synapse on GABAergic interneurons in the contralateral hemisphere (10). Accordingly, activation of a region in the ipsilateral hemisphere can lead to an inhibition of its contralateral homolog (11). This increases functional hemispheric asymmetries (FHAs), namely, differences in activation and dominance for different aspects of task processing between the hemispheres (12). For example, grammar and semantic aspects of language processing are predominantly processed by the left hemisphere (13) while face processing (14, 15) and visuospatial attention mainly rely on the right hemisphere (16).

Whether ipsilateral activation leads to inhibition or excitation in the contralateral hemisphere is influenced by factors like the specific task and exact location of activation (17, 18). Different steroid hormones can also influence the function of the corpus callosum (19): by interacting with glutamatergic and GABAergic transcallosal signaling, progesterone and estradiol can lead to a decrease in interhemispheric inhibition and increased bilateral activation (20). While this decreases functional asymmetries, it increases interhemispheric integration by strengthening information transfer across the corpus callosum (21).

The most widely investigated stress hormones are the catecholamines adrenaline and noradrenaline as well as the glucocorticoid cortisol. The former are released in response to activation of the sympathetic branch of the autonomous nervous system (22). Their increase leads to changes in heart rate, blood pressure, and subjective stress feeling. Cortisol, on the other hand, is the end product of the hypothalamus-pituitary-adrenocortical (HPA) axis (23). Secretion of corticotropin-releasing hormone from the hypothalamus leads to the release of adrenocorticotropic hormone (ACTH) from the anterior pituitary. ACTH in turn prompts the adrenal cortex to release cortisol into circulation. Transfer of information through the corpus callosum could be affected by cortisol (24). Inhibition of contralateral areas through the corpus callosum relies on glutamatergic excitation of GABAergic interneurons. As cortisol has been shown to upregulate glutamatergic neurotransmission (25, 26), increased cortisol concentrations could lead to an increase in glutamatergic activation of inhibitory interneurons. This would lead to an increase in interhemispheric inhibition through transcallosal fibers and thus an increase in FHAs.

In recent studies of our group investigating the effect of acute stress on FHAs, we found no changes in asymmetries on the behavioral level (27). However, interhemispheric integration of information was improved as indicated by an increased across-field advantage in a Banich–Belger task (28) after stress exposure. Interestingly, another study showed that interhemispheric transfer of lexical information was faster after stress induction in a lexical decision task (29). This indicates that acute stress strengthens interhemispheric communication for language stimuli. In a follow-up study, we aimed to disentangle the role of cortisol and negative affect by administering hydrocortisone instead of a psychosocial stressor. The results showed no effect of hydrocortisone on FHAs or interhemispheric integration (30). This indicates that acute stress does not exert its influence on interhemispheric communication through cortisol alone or at least, this relationship does not follow a simple linear dose-response curve. As there has not been any research on the effect of hydrocortisone administration on interhemispheric transfer of information, the current study aims at investigating this association. Similar to the study of interhemispheric integration after stress induction (29), we asked participants to perform a Poffenberger Paradigm (31) as well as a lexical decision task (32).

The EEG version of the classical Poffenberger paradigm can be used to estimate the transmission properties of the corpus callosum (33). Visual stimuli are presented in the left and right visual field; Latency differences in the N1 event-related potential between the left and right hemisphere for each stimulus are known as the interhemispheric transfer time (ITT). This is the time needed for the signal from the visual cortex contralateral to the visual field of stimulus presentation to travel across the corpus callosum to the contralateral homologous area (34). The N1 is an event-related EEG potential that consists of a negative deflection about 170 ms after stimulus onset. The component has been associated with the orientation of attention toward a stimulus (35).

Like the Poffenberger paradigm, the lexical decision task measures communication between the hemispheres. It can be used to investigate information transfer of lexical stimuli from one hemisphere to the other: language processing is primarily supported by the left hemisphere (36), which is reflected by shorter N1 latencies from language stimuli presented to the right visual half-field (37). Word stimuli presented to the left visual field are processed in the right hemisphere but also cross over to the left hemisphere as indicated by increased transcallosal connectivity for these stimuli (38).

If hydrocortisone has a similar effect on transcallosal transmission of information as acute stress on the neural level, we can expect faster information transfer in the lexical decision task from the left to the right hemisphere indicated by shorter latencies CP3-CP4 electrode pair after hydrocortisone administration compared to placebo. While our previous results (29) did not show an effect of stress or cortisol in the Poffenberger paradigm, we still chose to include this task to make a direct comparison with cortisol increases due to acute stress induction possible.

## 2. Materials and methods

### 2.1. Participants

A total of 50 male participants aged between 18 and 33 years (*M* = 24.7 Years, SD = 3.63) were part of this study. We chose to only test male participants as previous studies have shown that cycling phase-dependent changes in hormones can affect stress responsivity (39) and hemispheric asymmetries (20). The sample size was determined using *a priori* power analysis (G*power 3.1)^1^ with an α-error probability of 0.05 and a power of 0.95. Based on data by Brüne et al. (8), who investigated the effect of acute stress on hemispheric asymmetries, we estimated the effect of cortisol on hemispheric asymmetries to be small (partial η^2^ = 0.07). Inclusion criteria were an absence of mental or neurological disorders, no intake of medication or drugs, no smoking, and a healthy body mass index in the normal range (18.5–25 kg/m^2^). Additionally, participants must not perform shiftwork (40–42). Handedness was assessed using the Edinburgh Handedness Inventory (43). Six participants were left-handed, as categorized by a Lateralization Quotient (LQ) >0, and 44 were right-handed (*M* = 67.63, SD = 48.16). To better represent the typical distribution of hemispheric asymmetries in the population (44), we chose to include left-handed participants. The local ethics committee of the Ruhr University Bochum approved the study. Before the beginning of the first testing session, subjects gave written informed consent to participate. All participants were treated in accordance with the Declaration of Helsinki.

### 2.2. Procedure

**FIGURE 1:**
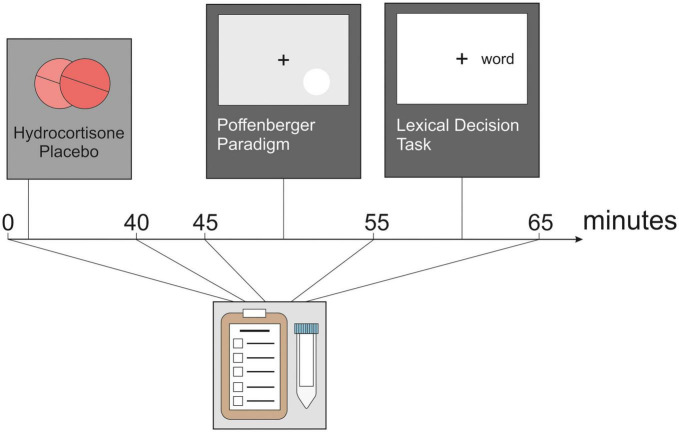
Experimental design. After administration of hydrocortisone or placebo, the participants completed a 5 min resting-state EEG as well as a Poffenberger paradigm and a lexical decision task. Before hydrocortisone administration and after each section of the experiment, cortisol, and affect are assessed.

Participants took part in two test sessions which took place between 2 and 6 p.m. at the Ruhr University Bochum, Germany, to minimize variance in cortisol data due to circadian changes in cortisol (42). After providing written informed consent, participants completed baseline subjective stress measurements and the first saliva sample was taken. Subjective stress was assessed with the Subjective Experiences Rating Scale [SERS; (45)]. Participants were given either two tablets of 10 mg hydrocortisone (Jenapharm^®^, Jena; Germany) each or a placebo. The dosage of 20 mg has been used in previous studies by our group and showed an influence on learning and memory processes (46, 47). Hydrocortisone and placebo conditions were pseudo-randomized between participants. Subsequently, participants waited 40 min before proceeding with the experiment. This time was used to set up participants with the EEG cap. Following this, participants underwent a 5 min resting state EEG recording and performed two tasks measuring information transfer across the corpus callosum in a pseudorandom order. Between these tasks, saliva samples were collected. In parallel, we assessed the effect of the participants (see Figure 1). Salivary samples were collected using Salivette sampling devices (Sarstedt, Nümbrecht, Germany).

### 2.3. Experimental paradigms

Paradigms were presented to participants using Presentation software (Neurobehavioral Systems, Albany, CA, USA). Participants placed their chin on a chinrest 57 cm from the computer screen. Participants were asked to always look at the fixation cross with a size of 1° by 1° visual angle at the center of the screen.

#### 2.3.1. Lexical decision task

For each trial, a stimulus was presented for 160 ms at a distance of 2° visual angle from the fixation cross to the left or right side (32). The intertrial interval (ITI) was jittered between 150 and 350 ms. Stimuli consisted of 80 German nouns as well as 80 pronounceable meaningless letter combinations that were created by exchanging two or more letters within the word. All stimuli were presented horizontally in a randomized order in black letters against white background with half in the left visual field (LVF) and the other half in the right visual field (RVF). Participants were asked to indicate via button press with their dominant hand if they believe to have seen a word or a non-word. Reaction time was limited to 2,000 ms.

#### 2.3.2. Poffenberger EEG paradigm

Each trial starts with the presentation (0.135 s) of a circular white stimulus (75.02 cd/m^2^) on a gray background (20.20 cd/m^2^) with a diameter of 1.41° with the outer edge of the stimuli at 5° horizontal and 5° vertical distance from the fixation cross to the lower left or right side of the fixation cross (48). To avoid expectancy effects, the intertrial interval (ITI) was jittered between 1,000 and 2,000 ms. Participants are asked to press a button as soon as they perceive the stimulus with the left or right hand. There was one block for each hand consisting of 25 left-sided and 25 right-sided presentations in a randomized order so there were 100 trials in total.

### 2.4. EEG recording and analysis

We recorded EEG with a 64 Ag–Ag Cl electrode system (actiCAP ControlBox and QuickAmp amplifier Brain Products GmbH, Gilching, Germany). Electrodes were positioned at standard scalp locations in accordance with the International 10–20 system (FCz, FP1, FP2, F7, F3, F4, F8, FC5, FC1, FC2, FC6, T7, C3, Cz, C4, T8, TP9, CP5, CP1, CP2, CP6, TP10, P7, P3, Pz, P4, P8, PO9, O1, Oz, O2, PO10, AF7, AF3, AF4, AF8, F5, F1, F2, F6, FT9, FT7, FC3, FC4, FT8, FT10, C5, C1, C2, C6, TP7, CP3, CPz, CP4, TP8, P5, P1, P2, P6, PO7, PO3, POz, PO4, and PO8). We used the FCz as online reference during recording. The sampling rate was 1 kHz and impedances were kept under 5 kΩ at the beginning of the experiment.

We used Brain Vision Analyzer software (Brain Products GmbH) for offline data analysis. First, we performed visual data inspection to reject EEG sections containing technical artifacts and to exclude faulty or dead channels. Data were filtered with a 1 Hz low-cut-off and a 30 Hz high cut-off filter (8 dB/oct). We applied semiautomatic independent component analysis (ICA) with Infomax rotation (49) to dispose of reoccurring artifacts like pulse artifacts, blink artifacts, and eye movement artifacts. Next, the FCz and all missing or rejected channels were interpolated using topographical interpolation with spherical splines. In the Poffenberger paradigm, data were epoched into stimulus-locked segments starting 100 ms before and 600 ms after stimulus onset. In the LDT, epochs extend from 200 ms prior to stimulus onset to 1,000 ms post-stimulus onset. We applied automatic artifact rejection with an allowed maximum voltage step of 50 μV/ms, a maximum value difference of 200 μV within a 200 ms interval or amplitudes below 0.1 μV. The number of trials rejected by this procedure was lower than 5% for all participants. Data were re-referenced using a CSD-transformation (50) to eliminate the reference potential from the data. After the CSD-transformation, epochs were baseline corrected and N1 amplitudes and latencies were averaged for all conditions for each participant individually.

For all further analyses, only correct trials were included. The N1 [130–230 ms after stimulus presentation, (51)] amplitudes and latencies were quantified at O1-O2 electrodes for the Poffenberger paradigm as they are positioned above the primary visual areas. For the Lexical Decision task, we used the CP3-CP4 electrodes as they are situated above Wernicke’s area.

### 2.5. Endocrinological measurements

To assess the effectiveness of hydrocortisone administration, salivary cortisol and salivary alpha-amylase activity were measured at five time points across the experiment (Figure 1): before the start of the experiment, participants gave a baseline salivary sample followed by four samples after 40, 45, 55, and 65 min. Salivary alpha-amylase (sAA) was used as a marker for sympathetic nervous system activity (52). Samples were stored at-20°C until analysis. Saliva samples were first 20 × diluted. Salivary cortisol was analyzed on a Synergy2 plate reader (Biotek, Winooski, VT, USA) using a commercial enzyme-linked immunosorbent assay (ELISAs; free cortisol in saliva; IBL/Tecan, Hamburg) according to the manufacturer’s instructions. Intra- and interassay variability of the assay was less than 10%. A colorimetric test using 2-chloro-4-nitrophenyl-α-maltotrioside (CNP-G3) as a substrate reagent was applied to measure salivary alpha-amylase activity as described elsewhere (53) and had an intra- and interassay variability of less than 15 and 10%, respectively.

### 2.6. Statistical analysis

We performed 2 × 5 repeated-measures ANOVAs with the factors treatment (hydrocortisone, placebo) and time point of measurement (1–5) for cortisol, salivary alpha amylase and affect.

#### 2.6.1. Lexical decision task

To investigate influences of hydrocortisone on transfer of lexical information in the LDT, we calculated repeated-measures ANOVAs with the factors treatment (hydrocortisone, placebo), visual field (LVF vs. RVF), hemisphere (left vs. right) and condition (word vs. non-word) for responses and reaction times of the behavioral LDT data. We performed the same analyses for EEG N1 latencies and amplitudes.

#### 2.6.2. Poffenberger paradigm

We chose to forgo analysis of behavioral data of the Poffenberger paradigm as these data are not reliable indicators for cortical information transfer (48). Due to signal transfer through subcortical pathways, it cannot be used to extrapolate from behavioral latency differences between left- and right-hand reaction latency to latency differences in the cortex (54).

For the EEG data, we calculated interhemispheric transfer times (ITT) in the Poffenberger paradigm by subtracting contralateral from ipsilateral latencies:


ITT⁢(LVF)=mean⁢(RH⁢_⁢LVF)-mean⁢(LH⁢_⁢LVF)

ITT⁢(RVF)=mean⁢(LH⁢_⁢RVF)-mean⁢(RH⁢_⁢RVF)

ITT=mean⁢(ITT⁢LVF,ITT⁢RVF)

In later analysis, we only used participants displaying a positive average ITT in the Poffenberger paradigm. We did so as a negative ITT is physiologically not possible: the signal from the stimulus enters the hemisphere contralateral to the side of presentation first due to the crossing of the optic fibers. From there, it transfers to the ipsilateral hemisphere resulting in a larger latency at ipsilateral electrode sites. A negative ITT could indicate, that participants did not keep their eyes focused on the fixation cross but made eye movements toward the stimuli negating the half-field presentation of the stimuli. This resulted in 42 participants for the Poffenberger paradigm.

To determine any differences in ITT between the hydrocortisone and placebo treatment, we computed a repeated measures ANOVA with the factors treatment (hydrocortisone, placebo), visual half-field (LVF vs. RVF) and electrode (O1 vs. O2) as well as a dependent sample *t*-tests comparing the total transfer time between sessions. All *post-hoc* tests were Bonferroni corrected.

## 3. Results

### 3.1. Cortisol administration

**FIGURE 2:**
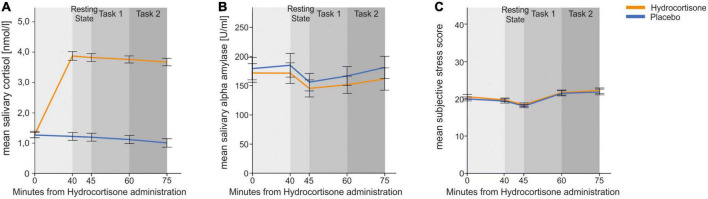
Physiological and subjective endocrinological and subjective response to hydrocortisone and placebo administration. Error bars represent 1 ± standard error of the mean (SEM). The first measurement was taken before tablet administration. **(A)** Mean cortisol (log-transformed in the figure only for presentiveness) **(B)** mean salivary alpha-amylase and **(C)** mean subjective stress responses measured by SERS for each time point.

For cortisol (see Figure 2A), we found a significant main effect of treatment [*F*(1,45) = 54.55, *p* < 0.001, η_p_^2^ = 0.55] and time [*F*(4,180) = 18.47, *p* < 0.001, η_p_^2^ = 0.29]. There was also a significant interaction effect of both [*F*(4,180) = 15.39, *p* < 0.001, η_p_^2^ = 0.26] indicating that administration of hydrocortisone lead to a significant increase in salivary cortisol. Bonferroni corrected *post-hoc* tests revealed that cortisol levels were increased for all measurement time points after hydrocortisone administration (all *p’s* < 0.001) compared to measurement time points after placebo administration.

We repeated the identical analysis for salivary alpha-amylase (see Figure 2B) to identify whether similar effects could be seen in the sympathetic nervous system. We found a significant main effect of time point of measurement [*F*(4,188) = 4.51, *p* = 0.002, η_p_^2^ = 0.08]. Pairwise comparisons revealed that sAA at the third time point was lower than at time point one (*p* = 0.038) and two (*p* = 0.001).

Lastly, we conducted the same analysis for affect measurement using the SERS (see Figure 2C). We found a significant main effect of time point [*F*(4,196) = 28.77, *p* < 0.001, η_p_^2^ = 0.37]. Pairwise comparisons revealed that SERS scores at the second and third time point were lower than at time point one, four and five (*p* < 0.001).

### 3.2. Behavioral data

#### 3.2.1. Lexical decision task

**FIGURE 3:**
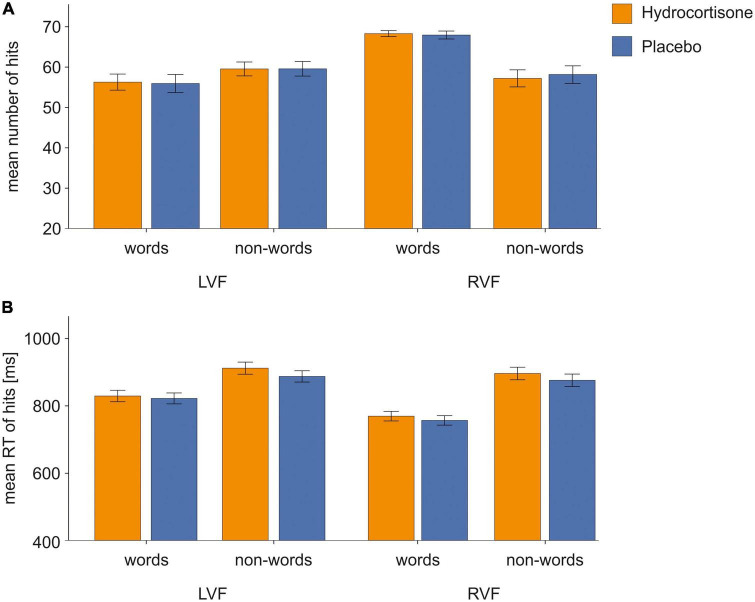
Behavioral data of correct responses in the lexical decision task. Error bars represent 1 ± SEM. For words, we found more correct responses and faster reaction times to stimuli presented in the right visual field indicating typical left-hemispheric dominance for language processing. There were no effects of hydrocortisone administration. **(A)** Number of correct responses and **(B)** reaction times of correct responses in the LDT.

We performed a repeated-measures ANOVA for the number of correct responses with the factors treatment (hydrocortisone vs. placebo), visual half-field (LVF vs. RVF) and condition (word vs. non-word) (Figure 3A; for descriptive data see Supplementary Table 1). The analysis revealed a significant main effect of visual half-field [*F*(1,49) = 41.18, *p* < 0.001, η_p_^2^ = 0.46; see Supplementary Table 2]. There was no main effect of hydrocortisone administration (*p* = 0.927). Moreover, there was a significant interaction of visual half-field and condition [*F*(1,49) = 40.17, *p* < 0.001, η_p_^2^ = 0.45]. A Bonferroni corrected *post-hoc* test revealed that participants reported more correct responses to stimuli presented in the right visual half-field than to stimuli presented in the left visual half-field (*p* < 0.001).

The repeated-measures ANOVA with the same factors for the number of incorrect responses demonstrated a significant main effect of visual half-field [*F*(1,49) = 40.43, *p* < 0.001, η_p_^2^ = 0.45; for descriptive data see Supplementary Table 1]. There was no main effect of hydrocortisone administration (*p* = 0.776; see Supplementary Table 2). Further, there was a significant interaction between visual half-field and condition [*F*(1,49) = 38.66, *p* < 0.001, ηp^2^ = 0.44]. A Bonferroni corrected *post-hoc* test revealed more incorrect responses to words presented in the left visual half-field compared to words presented in the right visual half-field (*p* < 0.001).

The analysis with the same factors for missed responses only revealed a significant interaction effect of condition and visual half-field [*F*(1,49) = 4,38, *p* < 0.05, η_p_^2^ = 0.08; for descriptive data see Supplementary Table 1]. This effect did not remain significant in a Bonferroni corrected *post-hoc* test (*p* = 0.058; Supplementary Table 2).

The repeated-measures ANOVA with the factors treatment (hydrocortisone vs. placebo), visual half-field (LVF vs. RVF) and condition (words vs. non-words) for reaction times of correct responses (for descriptive data see Supplementary Table 3) showed a significant main effect of condition [*F*(1,49) = 60.08, *p* < 0.001, η_p_^2^ = 0.55] and visual half-field [*F*(1,49) = 88.32, *p* < *0.001*, η_p_^2^ = 0.64] (Figure 3B and Supplementary Table 4). There was no main effect of hydrocortisone administration (*p* = 0.103). Moreover, there was a significant interaction between the factors condition and visual half-field [*F*(1,49) = 21.83, *p* < 0.001, η_p_^2^ = 0.31]. A Bonferroni corrected *post-hoc* test revealed faster responses to stimuli presented in the right visual half-field in the word condition compared to non-words (*p* < 0.001).

### 3.3. EEG data

#### 3.3.1. Lexical decision task

**FIGURE 4:**
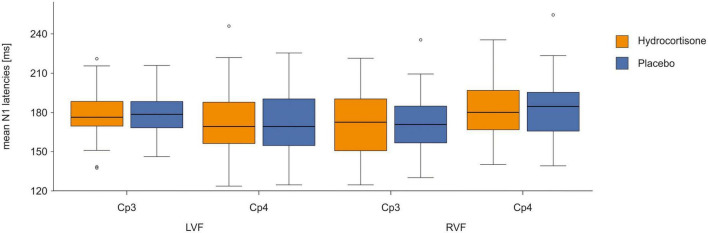
N1 latencies at the CP3/CP4 electrode pair after hydrocortisone and placebo administration.

To identify differences in latencies between the control and experimental condition, we performed a repeated-measures ANOVA with the factors treatment (hydrocortisone vs. placebo), visual field (left vs. right), electrode (CP3 vs. CP4), and condition (word vs. non-word) for N1 latencies (for descriptive data see Supplementary Table 5). The analysis showed no significant main effects of treatment, visual field, electrode or condition (all *p*s > 0.204; see Figure 4 and Supplementary Table 6). There was a significant interaction between electrode and visual field [*F*(1,49) = 9.31, *p* = 0.004, η_p_^2^ = 0.16]. Bonferroni corrected *post-hoc* tests revealed that latencies at the CP4 electrode were shorter for stimuli presented in the left visual field compared to the right visual field (*p* = 0.017). For latencies at the CP3 electrode, we observed the opposite effect with shorter latencies for stimuli presented in the right visual field, which was at trend level (*p* = 0.056). Moreover, there was a four-way interaction between treatment, visual field, electrode and condition [*F*(1,49) = 4.27, *p* = 0.044, η_p_^2^ = 0.08]. Bonferroni corrected *post-hoc* tests revealed shorter latencies at the CP3 electrode compared to the CP4 electrode for words in the right visual field after hydrocortisone administration (*p* = 0.013) and for non-words in the right visual field after placebo administration (*p* = 0.001).

**FIGURE 5:**
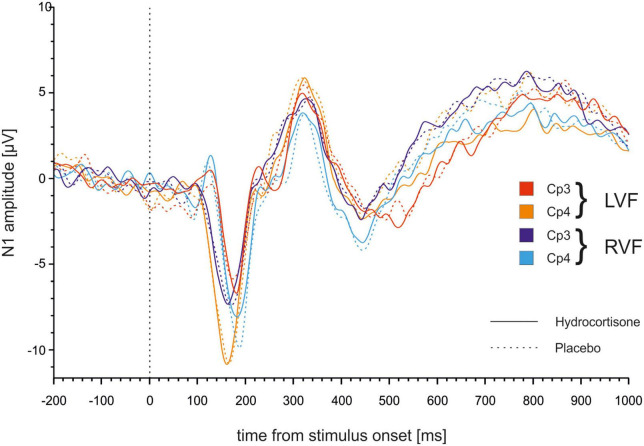
Time course of N1 ERPs at electrodes CP3 and CP4. Stimulus presentation was at 0 ms.

We performed the analysis with the same factors as before for amplitudes of the N1. The repeated measures ANOVA for amplitudes revealed a significant main effect of electrode [*F*(1,49) = 12.49, *p* = 0.001, η_p_^2^ = 0.20] indicating more negative amplitudes at the CP4 compared to the CP3 electrode (see Figure 5). Furthermore, the analysis demonstrated a significant interaction between electrode and visual field [*F*(1,49) = 13.98, *p* < 0.001, η_p_^2^ = 0.22]. Bonferroni corrected *post-hoc* tests revealed significantly more negative amplitudes for stimuli presented in the right compared to the left visual field at the CP3 (*p* = 0.005) while the opposite effect was evident at the CP4 (*p* = 0.17).

#### 3.3.2. Poffenberger paradigm

**FIGURE 6:**
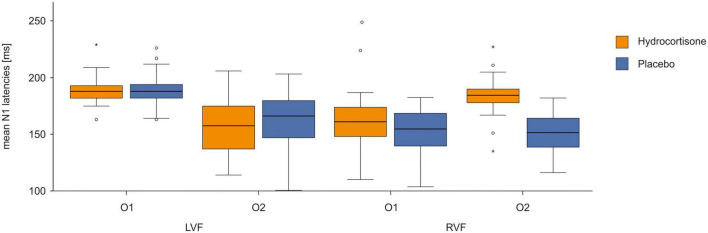
N1 latencies at the O1/O2 electrode pair after hydrocortisone and placebo administration.

To identify differences in latencies between the control and experimental condition, we performed a repeated measures ANOVA for N1 latencies with the factors treatment (hydrocortisone vs. placebo), visual half-field (left vs. right) and electrode (O1 vs. O2). We only included participants with a positive ITT leading to 42 participants for this analysis, as a negative ITT is physiologically not possible; this is indicative of participants likely failing to fixate on the fixation cross throughout the experiment (for descriptive data see Supplementary Table 6). The analysis revealed no significant main effects but a significant interaction between electrode and visual field [*F*(1,41) = 146.62 *p* < 0.001, η_p_^2^ = 0.78; for descriptive data see Supplementary Table 7]. A Bonferroni corrected *post-hoc* test revealed significantly shorter latencies at the O1 for stimuli presented in the right visual field as well as at the O2 for stimuli presented in the left visual field (*p* < 0.001, see Figure 6). Additionally, we found a significant interaction between treatment and visual field [*F*(1,41) = 6.96 *p* = 0.012, η_p_^2^ = 0.15]. A Bonferroni corrected *post-hoc* test revealed faster latencies for stimuli presented in the right visual field under placebo compared to stimuli presented under hydrocortisone (*p* = 0.005). A *t*-test revealed no significant differences between total transfer times between the sessions [*t*(41) = 0.192, *p* = 0.849] on the latencies of the N1 at the O1-O2 electrode pair.

**FIGURE 7:**
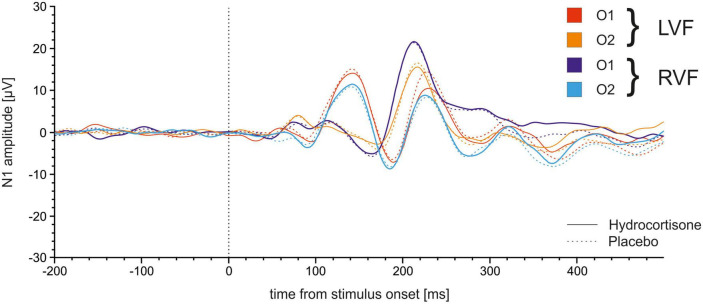
Time course of N1 ERPs at electrodes O1 and O2 in the Poffenberger paradigm. Stimulus presentation was at 0 ms.

We repeated the analysis with the same factors for amplitudes of the N1 (see Figure 7). No main effects or interactions reached significance (all *p*s > 0.169).

## 4. Discussion

The aim of the present study was to investigate the influence of administration of hydrocortisone on interhemispheric transfer of information. To this end, participants performed a Poffenberger paradigm as well as a lexical decision task twice, once after administration of hydrocortisone and once after administration of placebo. The administration of hydrocortisone successfully elicited a selective increase in salivary cortisol without influencing the level of salivary alpha-amylase or subjective stress. This gives rise to the opportunity of investigating the effects of cortisol on interhemispheric interaction specifically without the influence of other stress hormones or changes in emotional state. During placebo treatment, participants displayed the expected results in the Poffenberger paradigm and the lexical decision task. In the Poffenberger paradigm, we found shorter latencies at electrodes contralateral to the visual field of presentation compared to ipsilateral electrodes reflecting the time it takes for the signal to cross to the other hemisphere.

In the lexical decision task, we found shorter latencies for contralateral compared to ipsilateral presentation of lexical stimuli similar to studies before (32, 37). We did not find an effect of treatment that survived Bonferroni correction. We could replicate the classical Poffenberger effect from the literature with shorter latencies in the hemisphere contralateral to the stimulus presentation compared to the ipsilateral hemisphere (55). Additionally, we found slower latencies for stimuli presented in the right visual field after hydrocortisone administration.

Interestingly, studies from our group only found an association between cortisol and interhemispheric interaction when stress induction was employed and not in the studies that used pharmacological administration of hydrocortisone (30). Acute stress could affect interhemispheric interaction through other stress mediators than cortisol. For example, catecholamines and other factors of the fast-acting sympathetic nervous system may be essential for the possible impact of stress. Studies on the influence of cortisol on memory have demonstrated that sympathetic activity is necessary for cortisol to exert its effects on memory consolidation (56). Similarly, the presence of catecholamines might be necessary for possible effects of cortisol on FHAs and interhemispheric integration through the corpus callosum. Here, asymmetry and interhemispheric integration of information are akin to two sides of the same coin (21): while interhemispheric integration is reflected by less independent collaboration of the hemispheres, FHAs arise by more independent activity.

In the current study, we found increased latencies to stimuli presented in the right visual field after the administration of hydrocortisone in the Poffenberger task. This could indicate that hydrocortisone slowed processing of stimulus material from the right field of vision. As this interaction was independent of the hemisphere, it could be speculated that administration of hydrocortisone shifted attention away from the right visual field and thus slowed processing of stimuli presented on the right. Stimuli presented in the right visual field are primarily processed in the left hemisphere. As the right hemisphere has been suggested to be dominant for regulating the HPA axis and thus cortisol secretion (57–59), it could be speculated that administration of hydrocortisone had a stimulating effect on the right hemisphere leading to comparably slower processing on the left. This is in line with a previous study that demonstrated that cortisol lead to an increase in right frontal ERP voltage indicative of activation of the right superior frontal gyrus compared to the left (60). However, we did not find a similar effect in the lexical decision task: here, we found a four-way interaction with shorter latencies at the CP3 electrode compared to the CP4 electrode for words in the right visual field after hydrocortisone administration and for non-words after placebo administration. This is opposed to the effect in the Poffenberger paradigm, as it is indicative of faster left-hemispheric processing. A potential reason for this difference could lie in the different stimulus material in each task. Hence, no definitive conclusion can be drawn.

Another possible reason for the apparent discrepancy with our earlier studies (27, 29) could be related to the difference of total cortisol increase between acute stress and hydrocortisone administration and its relationship to the glucocorticoid receptors. Cortisol can bind to two receptors: the mineralocorticoid receptor and the glucocorticoid receptor (61). The MR has a high affinity for cortisol and is already occupied under basal cortisol levels whereas the GR has a lower affinity for cortisol and is only activated after stress exposure (62).

**FIGURE 8:**
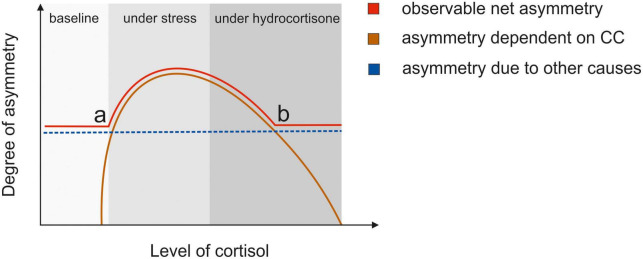
The hypothesized relationship between the degree of interhemispheric integration and level of cortisol. This model assumes that interhemispheric integration of information and functional hemispheric asymmetries can be seen as two sides of the same coin akin to assumptions made by Bayer et al. (21). The blue line indicates the level of asymmetry that is dependent on other factors than the corpus callosum, e.g., hemispheric specialization. This degree of asymmetry is constant and independent of levels of circulating hormones. The orange line indicates the degree of interhemispheric integration that depends on the corpus callosum, which can be influenced by changing hormone levels. If the cortisol level rises due to stress, the membrane MR receptors are partially occupied leading to a higher degree of integration. This leads to the apex in the curve. If cortisol levels are higher due to pharmacological administration of hydrocortisone, GR receptors are saturated. The red line indicates the observable degree of interhemispheric integration. At (a) the Integration rises due to rising cortisol levels improving callosal function. At (b) while the integration of information through the corpus callosum decreases due to high cortisol levels, the net integration levels out at the baseline degree of asymmetry that stays stable.

While acute stress leads to an increase in cortisol in the naturally possible range, the cortisol increase due to administration of hydrocortisone in the dosage used by us is much larger. It could be speculated that this affects the ratio of MR/GR occupation differently in studies using acute stress induction and hydrocortisone administration. As cortisol bind with a higher affinity to the MR, it is already saturated at lower cortisol levels (63). Thus, the higher cortisol levels due to pharmacological administration could disproportionately bind to the GR receptor. Furthermore, higher cortisol levels due to pharmacological administration could also target membrane-bound MRs, which have a lower affinity for cortisol than their cytoplasmic counterpart (64) with the result that membrane-bound MRs are only occupied under high cortisol levels. Thus, it is obvious that the pharmacological administration of hydrocortisone is not directly comparable to the physiological effects of moderate stress (see Figure 8).

Additionally, it needs to be mentioned that the effects of acute stress could not only be related to changes in stress hormone levels but also in other factors that accompany acute stress induction but not administration of hydrocortisone. For instance, increased negative affect and higher vigilance due to acute stress (24, 65) could contribute to the effects on interhemispheric integration. However, in our previous study, we did not find a relationship with affect (29). Moreover, effects of stress on hemispheric asymmetries might also only become apparent after chronic stress exposure or require early adversity to manifest (66).

### 4.1. Limitations and outlook

While the high temporal resolution of the EEG can allow for the investigation of fast processes like interhemispheric integration, it is not possible to specify the structural sources of the EEG due to the limited spatial resolution. This gives the opportunity for future studies to employ techniques with higher spatial resolutions such as fMRI to focus on the structural underpinnings in more detail elucidating the neural basis of interhemispheric integration (18).

An additional limitation concerns the sample of the current study. We only tested male participants. While this impedes the generalizability of our results, we chose to exclude women from this study due to the effects of cycling phase-dependent hormones on stress sensitivity (39) and hemispheric asymmetries (20, 67). Thus, any conclusions drawn from our data might not generalize to female participants as sex differences have been reported for lateralization as well as stress effects (68, 69).

## 5. Conclusion

In the present study, we found no changes in N1 latency or amplitude in the lexical decision task between hydrocortisone and placebo treatment. While we found increased latencies to stimuli presented in the right visual field in the Poffenberger task after the administration of hydrocortisone, this effect was not specific to one hemisphere and it is thus unlikely related to effects on the corpus callosum. This indicates that increases in cortisol levels alone are not sufficient to affect interhemispheric interaction through the corpus callosum. Furthermore, future imaging studies should employ methods with higher spatial resolution to further investigate the influence of stress hormones on the cooperation between the hemispheres and the influence on subcortical structures (70).

## Data availability statement

The raw data supporting the conclusions of this article will be made available by the authors, without undue reservation.

## Ethics statement

The studies involving human participants were reviewed and approved by the Local Ethics Committee of the Faculty of Psychology, Ruhr University Bochum (https://www.psy.ruhr-uni-bochum.de/psy/zentralebereiche/ethik.html.de). The patients/participants provided their written informed consent to participate in this study.

## Author contributions

OW and SO conceptualized and designed the study and provided feedback on the manuscript. GB collected the data, performed the analysis, and wrote the manuscript. JP wrote the manuscript. All authors contributed to the article and approved the submitted version.

## Footnotes

1
https://www.gpower.hhu.de/
