# Supplementary material for: A single dose of hydrocortisone does not alter interhemispheric transfer of information or transcallosal integration
Source: Front Psychiatry. 2023 Apr 18;14:1054168. doi: 10.3389/fpsyt.2023.1054168 (PMC10151494; doi:10.3389/fpsyt.2023.1054168)
Supplement: Supplementary file 1 [file Table_1.DOCX]

| Supplementary table 1: LDT Behavior | | | | | | |
| --- | --- | --- | --- | --- | --- | --- |
|  | Treatment | Condition | Visual Field | Mean | SD | N |
| Hit | Hydrocortisone | words | L | 56.28 | 14.09 | 50 |
|  |  |  | R | 68.28 | 5.23 | 50 |
|  |  | non-words | L | 59.54 | 12.19 | 50 |
|  |  |  | R | 57.20 | 14.98 | 50 |
|  | Placebo | words | L | 55.94 | 15.80 | 50 |
|  |  |  | R | 67.94 | 6.96 | 50 |
|  |  | non-words | L | 59.60 | 12.78 | 50 |
|  |  |  | R | 58.14 | 15.49 | 50 |
| Miss | Hydrocortisone | words | L | 1.04 | 2.25 | 50 |
|  |  |  | R | 0.64 | 1.76 | 50 |
|  |  | non-words | L | 0.90 | 2.35 | 50 |
|  |  |  | R | 0.94 | 2.25 | 50 |
|  | Placebo | words | L | 1.10 | 3.27 | 50 |
|  |  |  | R | 0.82 | 1.95 | 50 |
|  |  | non-words | L | 1.14 | 2.95 | 50 |
|  |  |  | R | 1.10 | 2.94 | 50 |
| Incorrect | Hydrocortisone | words | L | 22.68 | 13.92 | 50 |
|  |  |  | R | 11.08 | 4.70 | 50 |
|  |  | non-words | L | 19.56 | 12.31 | 50 |
|  |  |  | R | 21.86 | 15.14 | 50 |
|  | Placebo | words | L | 22.96 | 15.34 | 50 |
|  |  |  | R | 11.24 | 6.38 | 50 |
|  |  | non-words | L | 19.26 | 12.84 | 50 |
|  |  |  | R | 20.76 | 15.00 | 50 |

| Supplementary table 2: ANOVA LDT Behavior | | | | | |
| --- | --- | --- | --- | --- | --- |
|  |  | df | F | p | Partial η² |
| HitHit | Treatment | 1 | 0.01 | 0.927 | 0.00 |
|  | Condition | 1 | 3.88 | 0.055 | 0.07 |
|  | Visual Field | 1 | 41.18 | 0.000 | 0.46 |
|  | Treatment * Condition | 1 | 0.41 | 0.527 | 0.01 |
|  | Treatment * Visual Field | 1 | 0.43 | 0.517 | 0.01 |
|  | Condition * Visual Field | 1 | 40.17 | 0.000 | 0.45 |
|  | Treatment * Condition * Visual Field | 1 | 0.15 | 0.701 | 0.00 |
| Miss | Treatment | 1 | 0.53 | 0.471 | 0.01 |
|  | Condition | 1 | 1.52 | 0.223 | 0.03 |
|  | Visual Field | 1 | 1.82 | 0.184 | 0.04 |
|  | Treatment * Condition | 1 | 0.24 | 0.623 | 0.01 |
|  | Treatment * Visual Field | 1 | 0.02 | 0.890 | 0.00 |
|  | Condition * Visual Field | 1 | 4.38 | 0.042 | 0.08 |
|  | Treatment * Condition * Visual Field | 1 | 0.31 | 0.580 | 0.01 |
| Incorrect | Treatment | 1 | 0.08 | 0.776 | 0.00 |
|  | Condition | 1 | 3.61 | 0.063 | 0.07 |
|  | Visual Field | 1 | 40.43 | 0.000 | 0.45 |
|  | Treatment * Condition | 1 | 0.47 | 0.497 | 0.01 |
|  | Treatment * Visual Field | 1 | 0.46 | 0.502 | 0.01 |
|  | Condition * Visual Field | 1 | 38.47 | 0.000 | 0.44 |
|  | Treatment * Condition * Visual Field | 1 | 0.09 | 0.769 | 0.00 |

| Supplementary table 3: LDT Behavior Reaction Time | | | | | | |
| --- | --- | --- | --- | --- | --- | --- |
|  | Treatment | Condition | Visual Field | Mean | SD | N |
| Hit | Hydrocortisone | words | L | 829.10 | 120.65 | 50 |
|  |  |  | R | 769.15 | 100.83 | 50 |
|  |  | non-words | L | 911.89 | 127.05 | 50 |
|  |  |  | R | 896.04 | 132.32 | 50 |
|  | Placebo | words | L | 822.21 | 114.91 | 50 |
|  |  |  | R | 756.79 | 99.49 | 50 |
|  |  | non-words | L | 887.67 | 118.99 | 50 |
|  |  |  | R | 876.19 | 130.30 | 50 |
| Incorrect | Hydrocortisone | words | L | 931.29 | 173.79 | 50 |
|  |  |  | R | 938.26 | 179.98 | 50 |
|  |  | non-words | L | 914.99 | 178.06 | 50 |
|  |  |  | R | 816.55 | 128.27 | 50 |
|  | Placebo | words | L | 915.64 | 165.35 | 50 |
|  |  |  | R | 907.19 | 200.81 | 50 |
|  |  | non-words | L | 879.73 | 151.60 | 50 |
|  |  |  | R | 849.61 | 181.00 | 50 |

| Supplementary table 4: LDT Behavior Reaction Time | | | | | |
| --- | --- | --- | --- | --- | --- |
|  |  | df | F | p | Partial η² |
| Hit | Treatment | 1 | 2.76 | 0.103 | 0.05 |
|  | Condition | 1 | 60.08 | 0.000 | 0.55 |
|  | Visual Field | 1 | 88.32 | 0.000 | 0.64 |
|  | Treatment * Condition | 1 | 1.56 | 0.218 | 0.03 |
|  | Treatment * Visual Field | 1 | 0.01 | 0.934 | 0.00 |
|  | Condition * Visual Field | 1 | 21.83 | 0.000 | 0.31 |
|  | Treatment * Condition * Visual Field | 1 | 0.49 | 0.487 | 0.01 |
| Incorrect | Treatment | 1 | 0.66 | 0.422 | 0.01 |
|  | Condition | 1 | 16.30 | 0.000 | 0.25 |
|  | Visual Field | 1 | 18.05 | 0.000 | 0.27 |
|  | Treatment * Condition | 1 | 1.29 | 0.262 | 0.03 |
|  | Treatment * Visual Field | 1 | 1.86 | 0.179 | 0.04 |
|  | Condition * Visual Field | 1 | 6.72 | 0.013 | 0.12 |
|  | Treatment * Condition * Visual Field | 1 | 4.60 | 0.037 | 0.09 |

| Supplementary Table 5. LDT ERP | | | | | | | | |
| --- | --- | --- | --- | --- | --- | --- | --- | --- |
|  | Treatment | Electrode | visual field |  | Mean | SD | N |  |
| Latency | Hydrocortisone | CP3 | L | words | 179.12 | 26.79 | 50 |  |
|  |  |  |  | nonwords | 176.92 | 22.99 | 50 |  |
|  |  |  | R | words | 172.28 | 29.85 | 50 |  |
|  |  |  |  | nonwords | 171.80 | 29.72 | 50 |  |
|  |  | CP4 | L | words | 174.72 | 28.71 | 50 |  |
|  |  |  |  | nonwords | 171.90 | 24.71 | 50 |  |
|  |  |  | R | words | 184.16 | 19.48 | 50 |  |
|  |  |  |  | nonwords | 176.60 | 28.19 | 50 |  |
|  | Placebo | CP3 | L | words | 175.80 | 23.92 | 50 |  |
|  |  |  |  | nonwords | 179.68 | 22.31 | 50 |  |
|  |  |  | R | words | 175.16 | 26.90 | 50 |  |
|  |  |  |  | nonwords | 168.12 | 23.95 | 50 |  |
|  |  | CP4 | L | words | 174.18 | 27.52 | 50 |  |
|  |  |  |  | nonwords | 173.14 | 23.37 | 50 |  |
|  |  |  | R | words | 180.58 | 24.28 | 50 |  |
|  |  |  |  | nonwords | 183.76 | 26.22 | 50 |  |
| Amplitude | Hydrocortisone | CP3 | L | words | -11.20 | 8.77 | 50 |  |
|  |  |  |  | nonwords | -11.41 | 8.04 | 50 |  |
|  |  |  | R | words | -12.62 | 9.09 | 50 |  |
|  |  |  |  | nonwords | -14.71 | 11.04 | 50 |  |
|  |  | CP4 | L | words | -18.36 | 13.13 | 50 |  |
|  |  |  |  | nonwords | -17.19 | 13.44 | 50 |  |
|  |  |  | R | words | -13.84 | 9.00 | 50 |  |
|  |  |  |  | nonwords | -14.22 | 8.81 | 50 |  |
|  | Placebo | CP3 | L | words | -11.22 | 8.82 | 50 |  |
|  |  |  |  | nonwords | -11.40 | 8.01 | 50 |  |
|  |  |  | R | words | -13.06 | 8.67 | 50 |  |
|  |  |  |  | nonwords | -14.03 | 8.69 | 50 |  |
|  |  | CP4 | L | words | -18.19 | 14.14 | 50 |  |
|  |  |  |  | nonwords | -17.30 | 12.62 | 50 |  |
|  |  |  | R | words | -14.72 | 9.23 | 50 |  |
|  |  |  |  | nonwords | -14.51 | 9.12 | 50 |  |

| Supplementary Table 6. ANOVA of LDT ERP | | | | | |
| --- | --- | --- | --- | --- | --- |
|  |  | df | F | p | Partial η² |
| Latency | Treatment | 1 | 0.05 | 0.825 | 0.00 |
|  | Electrode | 1 | 0.82 | 0.369 | 0.02 |
|  | Visual Field | 1 | 0.17 | 0.685 | 0.00 |
|  | Condition | 1 | 1.66 | 0.204 | 0.03 |
|  | Treatment * Electrode | 1 | 0.22 | 0.639 | 0.01 |
|  | Treatment * Visual Field | 1 | 0.05 | 0.831 | 0.00 |
|  | Electrode * Visual Field | 1 | 9.31 | 0.004 | 0.16 |
|  | Treatment * Electrode * Visual Field | 1 | 0.12 | 0.735 | 0.00 |
|  | Treatment * Condition | 1 | 1.30 | 0.259 | 0.03 |
|  | Electrode * Condition | 1 | 0.05 | 0.827 | 0.00 |
|  | Treatment * Electrode * Condition | 1 | 2.99 | 0.090 | 0.06 |
|  | Visual Field * Condition | 1 | 0.70 | 0.407 | 0.01 |
|  | Treatment * Visual Field * Condition | 1 | 0.16 | 0.692 | 0.00 |
|  | Electrode * Visual Field * Condition | 1 | 0.55 | 0.461 | 0.01 |
|  | Treatment * Electrode * Visual Field * Condition | 1 | 4.27 | 0.044 | 0.08 |
| Amplitude | Treatment | 1 | 0.04 | 0.840 | 0.00 |
|  | Electrode | 1 | 12.49 | 0.001 | 0.20 |
|  | Visual Field | 1 | 0.47 | 0.496 | 0.01 |
|  | Condition | 1 | 0.28 | 0.598 | 0.01 |
|  | Treatment * Electrode | 1 | 0.19 | 0.668 | 0.00 |
|  | Treatment * Visual Field | 1 | 0.09 | 0.770 | 0.00 |
|  | Electrode * Visual Field | 1 | 13.98 | 0.000 | 0.22 |
|  | Treatment * Electrode * Visual Field | 1 | 0.24 | 0.626 | 0.01 |
|  | Treatment * Condition | 1 | 0.29 | 0.592 | 0.01 |
|  | Electrode * Condition | 1 | 3.61 | 0.063 | 0.07 |
|  | Treatment * Electrode * Condition | 1 | 0.13 | 0.724 | 0.00 |
|  | Visual Field * Condition | 1 | 3.08 | 0.086 | 0.06 |
|  | Treatment * Visual Field * Condition | 1 | 0.44 | 0.509 | 0.01 |
|  | Electrode * Visual Field * Condition | 1 | 0.05 | 0.830 | 0.00 |
|  | Treatment * Electrode * Visual Field * Condition | 1 | 0.01 | 0.914 | 0.00 |

| Supplementary Table 7. Poffenberger ERP | | | | | | |
| --- | --- | --- | --- | --- | --- | --- |
|  | Treatment | Electrode | visual field | Mean | SD | N |
| Latency | Hydrocortisone | O1 | L | 189.21 | 11.65 | 42 |
|  |  |  | R | 161.98 | 24.10 | 42 |
|  |  | O2 | L | 156.67 | 22.70 | 42 |
|  |  |  | R | 184.81 | 15.02 | 42 |
|  | Placebo | O1 | L | 188.95 | 12.89 | 42 |
|  |  |  | R | 153.14 | 19.30 | 42 |
|  |  | O2 | L | 161.57 | 24.32 | 42 |
|  |  |  | R | 182.19 | 16.61 | 42 |
| Amplitude | Hydrocortisone | O1 | L | -14.51 | 12.01 | 42 |
|  |  |  | R | -15.90 | 19.60 | 42 |
|  |  | O2 | L | -13.18 | 10.83 | 42 |
|  |  |  | R | -14.29 | 12.84 | 42 |
|  | Placebo | O1 | L | -11.96 | 11.59 | 42 |
|  |  |  | R | -14.42 | 14.70 | 42 |
|  |  | O2 | L | -11.34 | 13.38 | 42 |
|  |  |  | R | -13.44 | 12.59 | 42 |

| Supplementary Table 8. ANOVA of Poffenberger ERP | | | | | |
| --- | --- | --- | --- | --- | --- |
|  |  | df | F | p | Partial η² |
| Latency | Treatment | 1 | 0.95 | 0.336 | 0.02 |
|  | Electrode | 1 | 0.99 | 0.326 | 0.02 |
|  | Visual Field | 1 | 3.04 | 0.089 | 0.07 |
|  | Treatment * Electrode | 1 | 3.66 | 0.063 | 0.08 |
|  | Treatment * Visual Field | 1 | 6.96 | 0.012 | 0.15 |
|  | Electrode * Visual Field | 1 | 146.52 | 0.000 | 0.78 |
|  | Treatment * Electrode * Visual Field | 1 | 0.04 | 0.849 | 0.00 |
| Amplitude | Treatment | 1 | 1.35 | 0.252 | 0.03 |
|  | Electrode | 1 | 0.55 | 0.463 | 0.01 |
|  | Visual Field | 1 | 1.96 | 0.169 | 0.05 |
|  | Treatment * Electrode | 1 | 0.20 | 0.656 | 0.01 |
|  | Treatment * Visual Field | 1 | 0.35 | 0.557 | 0.01 |
|  | Electrode * Visual Field | 1 | 0.01 | 0.912 | 0.00 |
|  | Treatment * Electrode * Visual Field | 1 | 0.00 | 0.981 | 0.00 |
